# The Bacterial Role in the Progression of Breast Cancer through Mechanism of Gene Action: Future Prospects with Existing Studies

**DOI:** 10.2174/0113892029371937250918105052

**Published:** 2025-09-22

**Authors:** Yasir Nawaz, Saba Munir, Sidra Aslam, Fizza Rimal Butt, Fouzia Tanvir, Basit Nawaz, Kazam Bashir, Rikza Tahreem

**Affiliations:** 1 Jiangsu Key Laboratory for Microbes and Genomics, Department of Microbiology, School of Life Sciences, Nanjing Normal University, 1 Wenyuan Road, Nanjing 210023, China;; 2 Department of Zoology, Faculty of Life Sciences, University of Okara, Okara, Pakistan;; 3 Department of Biotechnology, Faculty of Sciences, University of Sialkot, Sialkot, Pakistan;; 4 Department of Chemistry, University of Agriculture Faisalabad, Faisalabad, Pakistan

**Keywords:** Breast cancer, microbiota, bacterial role, gene mechanism, cancer management

## Abstract

**Background:**

Breast cancer is the main cause of death for women, even with major improvements in treatment. Through processes like DNA damage, estrogen metabolism, and immunological regulation, bacterial populations have been shown to have an impact on breast cancer development in recent studies.

**Objectives:**

This review aimed to examine and evaluate current research on the involvement of bacteria in breast cancer progression, with an emphasis on gene action mechanisms and potential future treatments targeting the microbiome.

**Methods:**

A thorough literature analysis was carried out to identify pertinent research published between 1989-2024 across various databases, including PubMed, Google Scholar, Google, and Scopus.

**Results:**

Bacterial dysbiosis in the gut and breast tissue contributes to the progression of breast cancer through different pathways. Double-strand breaks in DNA are linked to various bacteria, like *Escherichia coli*, *Staphylococcus epidermidis*, *Helicobacter pylori,* and *Fusobacterium,* which contribute to genomic instability. Breast cancers are influenced by hormones that are influenced by gut microbiota, namely the estrobolome, which regulates estrogen levels. Bacteria also impact immune responses by preventing anti-tumor immunity. These results suggest that restoring microbial balance to specific bacterial taxa may open up new treatment options. Different genes may contribute to variations, including an increase in regulatory T (Treg) cells, while FOXP3+ T cells are linked to shorter relapse-free survival. Understanding the microbiota's role in DNA damage, hormone regulation, and immune modulation is important.

**Conclusion:**

Bacteria contribute significantly to the development of breast cancer through gene-level processes. Probiotics, immunomodulatory techniques, and microbiome-targeted treatments are potential future developments that could improve therapy effectiveness and reduce resistance.

## 1 INTRODUCTION

Cancer is distinguished by aberrant cells that spread and invade normal cells in the body. Breast cancer begins as a collection of cancer cells that originate in breast tissue and can eventually spread to other parts of the body or affect nearby cells. Since cells are the basic units that make tissue, that is where cancer starts [1]. When cell growth is disrupted, extra cells can sometimes form. When this occurs, the accumulation of cells often results in a mass of tissue known as a tumor or tissue lump [2, 3]. The human microbiome is made up of trillions of bacteria that inhabit diverse body regions, including the stomach and breast tissue [4]. According to recent research, the microbial communities found in breast cancers differ significantly from those present in nearby healthy tissue [5]. For example, studies have shown that some bacterial species, such as *Bacteroides fragilis*, are more common in breast tissue that has cancer than in those that do not have cancer [6]. *B. Fragilis* Toxin (BFT), a toxin produced by this bacterium, has been demonstrated to cause mammary gland epithelial hyperplasia, which aids in the development of tumors [7]. Bacteria cause breast cancer through a variety of mechanisms, the most common of which is chronic inflammation, which forms a cancer-friendly microenvironment [8]. Oxidative stress and DNA damage caused by pathogenic microorganisms can result in mutations that activate oncogenes and inhibit tumor suppressor genes [9]. Furthermore, bacterial interaction with hormone metabolism, namely estrogen, is critical in hormone-driven malignancies such as breast cancer. By altering hormone levels, some bacteria can affect estrogen pathways and increase the risk of cancer [10].

Numerous biological processes, such as inflammation, genetic mutations, hormone metabolism, and direct genetic connections, are believed to be involved in the association between bacteria and the development of breast cancer [11]. A major contributing factor to the development of cancer is chronic inflammation, which is frequently triggered by pathogenic bacteria like *Staphylococcus* and *E. coli* [12]. These bacteria can cause inflammation in breast tissue, which can result in DNA damage and oxidative stress [13]. Over time, this damage could lead to alterations that tilt the scales in favor of malignant transformation by suppressing tumor-suppressor genes or activating oncogenes [14]. Bacteria that interfere with estrogen pathways may directly contribute to the development and spread of breast cancer, as elevated estrogen levels are a known risk factor for the illness. Bacterial toxins directly affect genetic function in addition to inflammation and hormone metabolism. For instance, the toxin colibactin, which is produced by *E. coli*, can directly damage DNA and either cause or accelerate the development of tumors [15]. This demonstrates the intricate relationship between human genes and bacterial metabolites, whereby different species of bacteria can either stimulate or prevent the development of cancer [16]. Although previous studies have linked *H. pylori* to pancreatic and stomach cancers [17, 18], among other cancers, its precise function in breast cancer is still unclear. Newer studies, however, indicate that *H. pylori* might also be a major factor in the development of other cancers, such as breast cancer [19]. *H. pylori* may affect breast cancer development through various processes, including interactions with the immune system, changes to the tumor microenvironment, and the activation of oncogenic pathways [20]. Research has indicated correlations between *H. pylori* infection and increased tumor marker levels in patients with breast cancer, indicating a possible link that needs more investigation [21].

Through a variety of routes, such as the skin, gastrointestinal system, and oral cavity, bacteria colonize breast tissue and may increase the risk of breast cancer [10]. Through cuts or piercings, skin bacteria such as Staphylococcus epidermidis can penetrate deeper tissues, causing inflammation that fuels the growth of cancer [22]. While the gut microbiota, particularly the “estrobolome,” affects estrogen metabolism and hormone-driven malignancies, breastfeeding transfers both beneficial bacteria like *Lactobacillus* and pathogenic ones like *Staphylococcus aureus* into the mammary ducts [23]. Furthermore, lung infections and oral bacteria like *Porphyromonas gingivalis* can enter the bloodstream and go to breast tissue, where they can cause chronic inflammation and cancer [24]. The growing knowledge of the breast microbiome and how it interacts with pathways linked to cancer creates new opportunities for investigation and treatment approaches. Through processes like direct genetic damage, hormone metabolic disturbance, and persistent inflammation, bacteria aid in the development of breast cancer [25]. Finding the unique microbial fingerprints in individuals with breast cancer may result in the development of targeted treatments aimed at altering the bacterial composition of breast tissue, as well as new diagnostic techniques like microbiome profiling. Probiotics, antibiotics, and microbiome-targeted therapies should all be investigated further as potential components of individualized breast cancer preventive and treatment plans. The effects of these therapies are listed in their work. The bacterial role in breast cancer is shown in Fig. (**1**). This study highlights the bacterial role in breast cancer progression and the genomics variations causing breast cancer.

## 2 HISTORY OF CANCER

The history of cancer shows how mankind has understood and responded to this complicated illness across time, moving from traditional theories to more recent scientific research. Centuries of medical philosophy began with the first accounts in ancient Egypt and the fundamental ideas presented by Hippocrates and Galen [26]. The invention of the microscope during the Renaissance led to significant breakthroughs that allowed for a more in-depth investigation of cellular structures. The discovery of environmental carcinogens and the development of oncology as a distinct field during the 19^th^ and 20^th^ centuries marked momentous times, characterized by important turning points like the National Cancer Act and the establishment of the National Cancer Institute [27]. Modern advancements in immunotherapy, personalized medicine, and genetic research are transforming therapeutic modalities and providing promise for better results [28]. A journey through cancer history highlights human inventiveness and tenacity in the face of one of the greatest health challenges, despite continued hurdles. The history of cancer can be seen in Table **1**.

## 3 BREAST CANCER PREVALENCE

The World Health Organization (WHO) 2023 reports that breast cancer is the most prevalent cancer form among women globally [29]. Breast cancer's startling incidence and fatality rate make it a major worldwide health problem even now. Although genetics is a major element in its development, new research indicates that the microbiome, especially bacteria, may also be involved in its advancement. The incidence of breast cancer has been rising significantly in Pakistan in recent years, which is consistent with a global trend. Although the precise prevalence rates vary depending on the region and population, it is evident that breast cancer is a major health concern in Pakistan [30]. Table **2** presents the global prevalence of breast cancer in 2023, which provides comprehensive data on incidence, mortality, survival rates, 5-year prevalence, and the mortality-to-incidence ratio.

A thorough summary of breast cancer incidence, death, 5-year prevalence, survival rates, and mortality-to-incidence ratios in various global regions is provided by the breast cancer prevalence table. The incidence of breast cancer reached 2.30 million cases worldwide, with 0.67 million deaths and a 5-year prevalence of 8.50 million, indicating the disease's continued difficulty. Due to their advanced healthcare systems, early detection, and access to treatment, Europe and the Americas have the best survival rates, at 90% and 85%, respectively. Africa, on the other hand, has the greatest mortality-to-incidence ratio (0.57) and the lowest survival rate (70%) of any continent [33]. Early detection, awareness campaigns, and easier access to cancer treatments are factors that lower mortality rates (ratios of 0.13 and 0.25, respectively) in high-income regions like Europe and the Americas. In contrast, late-stage diagnosis, a lack of screening programs, and limited healthcare services contribute to Africa's noticeably higher mortality ratio [35]. In general, this table illustrates advancements as well as ongoing obstacles in the fight against breast cancer, especially in areas with less established healthcare systems [36].

## 4 BREAST CANCER GENOMICS

Despite advances in understanding the breast cancer genome, translating these insights into improved patient health remains a major issue. Enormously parallel sequencing, whether by targeted approaches like exome or candidate gene sequencing or unbiased WGS, has become a standard research tool [37, 38]. The cancer sequencing studies can identify genes with somatic mutations that drive malignant transformations. Genes that accumulate somatic mutations at a rate higher than expected by chance are known as “Significantly Mutated Genes” (SMGs) and play a role in cancer progression. In BC, the list of SMGs varies significantly between luminal-type and basal-like subtypes. Data from The Cancer Genome Atlas (TCGA) shows that luminal A BC has at least 20 SMGs, luminal B has 8, while basal-like breast cancer has only 3. However, this does not indicate that luminal BC genomes are more complex; in fact, basal-like BC genomes are often so structurally chaotic that identifying key mutations by recurrence statistics is challenging. Structural rearrangements such as large-scale chromosomal deletions, amplifications, inversions, and translocations are particularly significant in basal-like breast cancer [39].

Recently, many mutations can occur in a short time due to a catastrophic cell division in which extensive chromosomal breaks are (rarely) repaired in a viable manner. This process, known as chromothripsis, reshuffles the genome in a way that simultaneously amplifies oncogenes and deletes tumor suppressor genes, promoting rapid malignant transformation [40]. The reported frequency of chromothripsis in BC ranges from 2% to 11.06% [40, 41]. Given that both chromothripsis and interval BC share the characteristic of sudden onset, it is hypothesized that chromothripsis may underlie the rapid development of interval BC tumors that arise between routine screening visits. Because these tumors emerge within a short timeframe, traditional screening methods may be ineffective in detecting them early.

## 5 MATERIALS AND METHODS

The data was retrieved from different sources, including Google Scholar, PubMed, Google, and Scopus, to complete the study. The study included different cancers caused by various sources of bacteria, while others were excluded.

## 6 SOURCES OF BACTERIA

Breast tissue can become colonized by bacteria *via* a variety of entrance points and routes. Comprehending these origins is imperative for researching the part bacteria play in the advancement of breast cancer and their possible correlation with genes that impact the genesis of cancer [42]. The main places where bacteria can infiltrate or damage breast tissue are listed below:

### 6.1 Skin Microbiota

The skin is one of the most significant reservoirs of bacteria, and the breast, being an external organ, is in constant contact with skin microbiota. Bacteria such as *Staphylococcus epidermidis* and *Propionibacterium acnes* are commonly found on the skin and can potentially migrate to the breast tissue. Small wounds, surgical procedures, and nipple piercings are examples of skin integrity disruptions that may allow these germs to enter deeper tissues. Certain inflammatory processes and the local immune response are known to play a role in the development of cancer, and some research has suggested that the skin microbiome may have an impact on these processes [5].

Vitiligo is an acquired skin disorder characterized by melanocyte dysfunction and depigmentation. Significant alterations in the expression of melanocortin system genes in vitiligo-affected skin were found in a study [43]. They observed reduced expression of melanocortin receptors and melanogenesis enzymes in lesional skin, while, unexpectedly, melanocortin receptors were significantly upregulated in non-lesional skin. This increased receptor expression was accompanied by the upregulation of genes encoding melanin synthesis enzymes [44]. Melanogenesis in human melanocytes is regulated by multiple pathways, primarily the cyclic adenosine monophosphate/protein kinase A (cAMP/PKA) pathway, which transmits melanocortin system signals to key enzymes such as Tyrosinase (TYR), Tyrosinase-Related Protein-1 (TYRP1), and Dopachrome Tautomerase (DCT). Additionally, melanin synthesis is influenced by the Wnt and MAPK (mitogen-activated protein kinase) pathways, as well as the Inositol Phosphate/Protein Kinase C (IP3/PKC) and Nitric Oxide/Protein Kinase G (NO/PKG) pathways [45].

### 6.2 Breastfeeding and Mammary Gland Microbiota

Through the infant's oral flora, breastfeeding delivers a range of germs to the breast. Via the nipple, these bacteria can enter the mammary ducts and form colonies in the breast tissue. Breastfeeding facilitates the transfer of beneficial bacteria, like *Lactobacillus*, which help preserve tissue homeostasis. On the other hand, harmful bacteria like *Staphylococcus aureus*, which can cause mastitis, can worsen inflammation and foster environments that are favorable to the growth of cancer. According to recent research, the mammary gland has its own microbiome that interacts with the immune system and may have an impact on the risk of breast cancer [46].

### 6.3 Gastrointestinal Tract and Gut Microbiome

The gut microbiome is essential to overall health, and changes in the composition of gut bacteria have been linked to various illnesses, including breast cancer. A higher level of circulating estrogen is linked to a higher risk of estrogen-receptor-positive breast tumors due to the modulation of estrogen metabolism by certain gut flora. By encouraging hormone-driven cell proliferation in breast tissue, the so-called “estrobolome,” a subgroup of gut bacteria involved in estrogen metabolism, may have an impact on the development of cancer. Moreover, circulation may facilitate bacterial transfer from the stomach to other organs, such as the breast, enabling gut-derived germs to affect distant tissue settings [47].

### 6.4 Oral Microbiome

Another significant source of germs that may have an effect on breast health is the mouth cavity. A higher risk of systemic disorders, including cancer, has been connected to oral bacteria linked to periodontitis, such as *Porphyromonas gingivalis* and *Fusobacterium nucleatum*. Through the gums, these bacteria may enter the bloodstream and spread to other parts of the body, such as the breast. Once within the breast tissue, these bacteria may change the local immune response or cause chronic inflammation, which would accelerate the development of breast cancer [48].

### 6.5 Respiratory Tract Microbiome

Inhaled bacteria from the respiratory tract can also play a role in breast cancer development. The lungs are continuously exposed to bacteria from the environment, and breathing in some pathogens may cause them to spread through the bloodstream and throughout the body. Respiratory pathogens, particularly those associated with chronic infections or inflammatory conditions, may indirectly affect breast cancer risk by promoting systemic inflammation or immune dysregulation, both of which can create an environment conducive to cancer [49].

### 6.6 Environmental and External Sources

Bacteria from external and environmental sources, such as contaminated soil, water, or air, can come into contact with breast tissue. The breast microbiome can be changed by elements such as exposure to pollution, bacterial toxins, or bacteria resistant to antibiotics. By releasing toxins, modifying the immune system, or creating oxidative stress, these bacteria may have an impact on the development of breast cancer. These actions can all lead to DNA damage and carcinogenesis [50].

### 6.7 Hormonal Imbalance and Systemic Inflammation

Variations in estrogen levels, in particular, can impact the bacteria that comprise breast tissue. An imbalance in this microbiota can lead to high estrogen levels, which increases the risk of breast cancer. Certain bacteria, such as those in the gut estrobolome, metabolize estrogen. In addition to being a potential cause of chronic infections or immunological dysregulation, systemic inflammation can facilitate the movement of germs from other areas of the body, such as the skin, lungs, or gut, into the breast [50].

### 6.8 Mammary Microbiota and Breast Cancer

Given the varied impacts the microbiome has on different organs, recent research has shifted towards investigating the bacteria that inhabit breast tissue. Studies have found specific microbiota present in breast milk, and some researchers propose that bacteria may enter the breast ducts through the nipple, forming a unique microbiome within the breast. This is consistent with the idea that skin and oral bacteria can access the breast ducts *via* the nipple. However, interestingly, recent studies suggest that these bacteria may actually originate from the mother's gastrointestinal tract. They investigated the potential role of microbiota in ER+ Breast Cancer (BC) by sequencing the 16S ribosomal RNA (rRNA) in both breast tumor tissue and the adjacent healthy tissue from the same patients. They found that Methylobacterium radiotolerans was more abundant in the tumor tissue, while Sphingomonas yanoikuyae was more prevalent in the nearby healthy tissue. Furthermore, the overall bacterial DNA content was lower in tumor tissue compared to healthy adjacent tissue and was inversely associated with advanced disease stages. This finding has significant implications for the diagnosis and staging of BC. Additionally, they noted that antibacterial response genes were expressed at lower levels in tumor tissue compared to healthy breast tissue [51]. They discovered significant differences in the levels of Methylobacterium radiotolerance when comparing lymph node cancer samples to the adjacent normal tissues. In contrast, Wang *et al.* found a reduced relative abundance of Methylobacterium in invasive breast carcinoma compared to breast tissue from healthy women. However, they observed that cancer patients exhibited higher levels of Gram-positive organisms, such as *Corynebacterium, Staphylococcus, Actinomyces*, and *Propionibacteriaceae* [52, 53]. They reported that the most prevalent phyla in breast tissues were Proteobacteria, Actinobacteria, and Firmicutes. Proteobacteria were more abundant in breast tumor samples, while Actinobacteria dominated in the adjacent normal tissue. However, Meng *et al.*, using a cohort of Chinese patients, found an increased presence of the genus *Propionicimonas* and the families Micrococcaceae, Caulobacteraceae, Rhodobacteraceae, Nocardioidaceae, and Methylobacteriaceae in malignant breast tumor tissues. It is important to note that these findings may be influenced by the ethno-specific characteristics of the study population [54, 55]. Banerjee *et al.* reported that each Breast Cancer (BC) subtype has a distinct viral, bacterial, fungal, and parasitic profile. In their study, ER and HER2-positive BC subtypes exhibited similar patterns, while Triple-Negative Breast Cancer (TNBC) tissues displayed distinct characteristics. Proteobacteria were the primary signatures shared among all four subtypes, although signatures of Actinomyces were also detected. Additionally, Costantini *et al.* identified Proteobacteria, Firmicutes, Actinobacteria, and Bacteroidetes as being associated with breast tumors, with the Ralstonia genus being the most prominent among them.

They reported a markedly different breast microbiota pattern when comparing tumor and healthy breast tissue from women with Breast Cancer (BC) to tissue from healthy individuals. Women with BC showed higher relative abundances of Bacillus, Comamonadaceae, Bacteroidetes, Enterobacteriaceae, and Staphylococcus compared to healthy controls. Notably, Enterobacteriaceae and Staphylococcus are capable of causing DNA damage, including double-strand breaks, while Bacillus may contribute to carcinogenesis through hormone metabolism and stimulation of cell proliferation. Conversely, lower levels of bacteria with anti-cancer properties, such as Lactococcus and Streptococcus, were found in women with BC compared to healthy individuals. They observed significant differences in β-diversity (the variation in microbial communities between samples) when comparing the breast tissue microbiome of women with benign breast disease to those with invasive Breast Cancer (BC). The breast tissue of women with BC showed a significant enrichment of gender-specific taxa, including *Fusobacterium*, *Atopobium*, *Gluconacetobacter*, *Hydrogenophaga*, and *Lactobacillus*. Additionally, an analysis of the functional roles of these bacteria within the microenvironments revealed six differentially abundant pathways when comparing patients with benign *versus* malignant breast conditions. In BC patients, there was a notable overexpression of genes involved in cysteine and methionine metabolism, glycosyltransferases, and fatty acid biosynthesis [56]. Interestingly, dependence on methionine is a common metabolic issue observed in various cancers, and it is suggested that reducing methionine levels could potentially reverse cancer progression [57].

They noted that postmenopausal women with Breast Cancer (BC) exhibited altered fecal microbiota and reduced α-diversity (the variation of microbes within a single sample), which was independently linked to estrogen levels. They found that patients with BC had increased levels of Clostridiaceae, *Faecalibacterium*, and Ruminococcaceae, while levels of *Dorea* and Lachnospiraceae were lower compared to matched controls [58]. They found that postmenopausal women with Breast Cancer (BC) had fecal microbiota enriched with *Escherichia coli*, *Citrobacter koseri*, *Acinetobacter radioresistens*, *Enterococcus gallinarum*, *Shewanella putrefaciens*, *Erwinia amylovora*, *Actinomyces* spp. HPA0247, *Salmonella enterica*, and *Fusobacterium nucleatum*. However, no differences were observed between cases and controls in premenopausal women [59]. However, the findings from these various studies do not enable definitive conclusions due to differences in study populations regarding age, ethnicity, geographical location, sequencing techniques, and analysis methodologies.

## 7 MECHANISM OF ACTION: BACTERIAL INTERACTION WITH GENES IN BREAST CANCER PROGRESSION

**Fig. (2):**
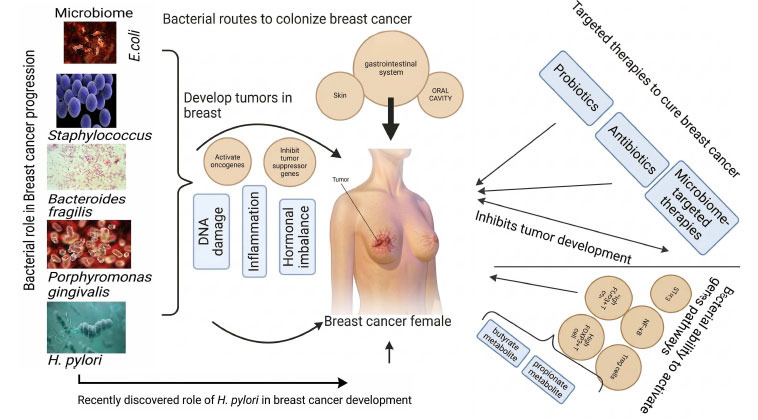
Shows the bacterial role and gene action in breast cancer development.

Breast Cancer (BC) was once regarded as a non-immunogenic tumor. However, recent studies have demonstrated that the expression of immune-related genes and the presence of immune infiltrates in primary tumors are linked to improved clinical outcomes. This finding is particularly noteworthy for HER2+ and Triple-Negative Breast Cancer (TNBC), which are the most aggressive subtypes. In this context, CD8+ T cells, which are typically cytotoxic T cells, can directly eliminate cancer cells, and their presence is associated with a better prognosis. Conversely, FOXP3+ CD4+ regulatory T cells primarily facilitate immune tolerance, and their presence correlates with a poorer prognosis. In Breast Cancer (BC), the percentage of regulatory T (Treg) cells increases with the progression of the disease, moving from normal tissue to *in situ* Ductal Carcinoma (DCIS) and from DCIS to invasive carcinoma. In patients with invasive carcinomas, a high number of FOXP3+ T cells is associated with shorter relapse-free survival and overall survival. This suggests the presence of Treg cells with immunosuppressive properties that facilitate immune evasion and promote cancer progression. Although the mechanisms by which growing tumors induce the proliferation and differentiation of Treg lymphocytes are not fully understood, it is believed that tumor cells produce prostaglandin E2, while tumor-associated macrophages secrete the cytokine CCL22, both of which can serve as chemotactic and differentiation factors for these cells [60]. It has been proposed that changes in the abundance of specific bacteria within the gut microbiota can lead to increased production of regulatory T cells (Tregs) or inhibit the differentiation of pathogenic T cells, potentially helping to prevent inflammatory diseases. For instance, Treg cells that express the FOXP3 transcription factor play a crucial role in modulating the immune response to commensal microbiota, and metabolites produced by these bacteria may influence Treg cell turnover. Additionally, certain bacterial metabolites, such as butyrate and propionate, have been shown to have strong anti-inflammatory effects by modulating colonic regulatory T cells in animal models [61, 62]. In another study, Goedert *et al.* found a significant estrogen-independent association between IgA+ and IgA− gut microbiota in Breast Cancer (BC) patients. When comparing BC patients with IgA+ and IgA− microbiota, those with IgA+ exhibited significantly lower richness and α-diversity in their fecal microbiota than those with IgA− microbiota. The estrogen-independent associations between IgA+ and IgA− gut microbiota showed notable differences when comparing controls and postmenopausal BC women, suggesting that gut microbiota may affect BC risk by influencing metabolism, estrogen recycling, and immune pathways. This is shown in Fig. **2**.

Studies examined smoking-induced molecular alterations and their impact on diseases [63, 64]. Research has identified an association between global DNA methylation and tobacco smoking in cancer-related tissues, though no such link was found in normal samples [65, 66]. A heatmap of gene expression data, based on the 50 genes with the lowest *P* values, revealed a distinct smoking-related expression pattern. Male smokers clustered into two groups with distinct gene expression profiles. The genes GPR15, RTKN2, USP46, CCR4, and CCR8 formed a cluster of upregulated genes. Notably, FOXP3 and GPR15 exhibited similar expression patterns, with a correlation coefficient of 0.59 (*P* = 1.15E-05). GPR15 plays a role in the homing of regulatory T cells (FOXP3^+^), and FOXP3 significantly binds to the GPR15 enhancer in humans [67]. Smoking induces a transcriptional network that promotes the upregulation of GPR15, reinforcing its functional relevance in smoking-induced molecular changes [68].

Collectively, these findings suggest that microbial DNA found in the breast, along with metabolites produced by bacteria, may impact the local immune microenvironment. This implies that our commensal bacteria could directly affect tumor processes through their metabolic capabilities, influencing immune cells and the inflammatory response.

### 7.1 Inflammation-Induced Gene Expression

Bacteria have the ability to cause long-term inflammation in breast tissue, which can activate signaling pathways, such as STAT3 and NF-κB. These pathways are known to support the survival, growth, and metastasis of cancer cells. In addition, pro-inflammatory genes are triggered by the production of cytokines, which contribute to the development of a microenvironment that is conducive to the spread of cancer [69].

### 7.2 DNA Damage and Mutagenesis

Some bacteria can create toxins that directly harm DNA, such as *E. coli genotoxins*. These bacteria are often linked to persistent infections. This mutagenesis raises the possibility of malignant changes in breast tissue by activating oncogenes or inactivating tumor suppressor genes [70].

### 7.3 Epigenetic Modifications

Histone modifications and DNA methylation are two epigenetic modifications that can be induced by bacterial metabolites. These alterations could speed up the development of cancer by silencing tumor suppressor genes or activating oncogenes. For instance, alterations in gene expression that affect the risk of breast cancer have been connected to the production of short-chain fatty acids by specific gut bacteria [71].

### 7.4 Immune System Modulation

It is possible for bacteria to either strengthen or weaken the immune system's capacity to identify and eradicate cancerous cells. Certain bacteria can lead to immunological tolerance, which makes it possible for cancer cells to avoid immune monitoring, while other bacteria can trigger immune reactions that cause tissue damage and persistent inflammation, both of which can accelerate the development of cancer [72]. This can be seen in Table **3**.

## 8 FUTURE PROSPECTS AND RECOMMENDATIONS

Due to its unique immune-activation properties and inherent tumor-targeting capabilities, bacterial-mediated anti-tumor therapy has garnered considerable attention. It has made great strides in overcoming the drawbacks of monotherapy and successfully eliminating malignancies, particularly when paired with more conventional forms of treatment like radiation. Bacteria and their derivatives exhibit a range of biological properties that allow them to both protect healthy tissues and enhance the sensitivity of tumor cells to radiation. Furthermore, the range of uses for microorganisms in radiotherapy has been further broadened by genetically modified bacteria and biomaterials derived from bacteria [79].

In the future, there is an urgent need to treat breast cancer promptly. As breast cancer is increasing on a daily basis, to cope with this alarming situation, there is a need for different therapies to cure it completely. Better screening and increased public awareness have led to early diagnosis at phases that can be treated with curative medicines and total surgical resection. The use of antibiotics may alter the risk of breast cancer. Only by taking such prompt action can the global burden of breast cancer be reduced. All women have equal access to medical care, from screening to sophisticated treatment. To determine the factors influencing the link popularity of websites related to breast cancer and to test the theory that more well-known websites are of higher quality, an online study survey should be conducted. There is an urgent global need to identify breast cancer in its early stages. Chemotherapy has evolved to encompass molecular abnormalities that are necessary for both targeted treatment and the identification of potential new medications.

## CONCLUSION

In conclusion, breast cancers may arise due to chromothripsis, a sudden genomic reshuffling that drives rapid tumor development. Since these cancers emerge too quickly for routine screening to detect, studying their genomic structure is crucial for improving patient outcomes. The evidence suggests that targeting bacterial dysbiosis and restoring microbial balance could be a promising approach in developing novel breast cancer therapies. Different bacteria play a role in breast cancer progression, including *Fusobacterium*, *Atopobium*, *Gluconacetobacter*, *Hydrogenophaga*, and *Lactobacillus*, *Corynebacterium, Staphylococcus, Actinomyces*, and *Propionibacteriacea,* as well as *Escherichia coli*, *Citrobacter koseri*, *Acinetobacter radioresistens*, *Enterococcus gallinarum*, *Shewanella putrefaciens*, *Erwinia amylovora*, *Actinomyces* spp. HPA0247, *Salmonella enterica*, and *Fusobacterium nucleatum*. At the genomics level, different genes contribute to variations in their effect, including an increase in regulatory T (Treg) cells, while FOXP3+ T cells are associated with shorter relapse-free survival. To manipulate the microbiota's role in DNA damage, hormone regulation, and immune modulation, breast cancer treatment and personalize therapeutic strategies can be improved.

## Figures and Tables

**Fig. (1):**
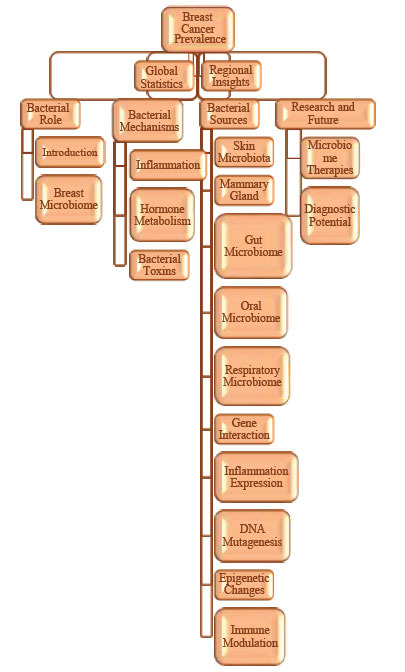
Flow chart diagram of the bacterial role in breast cancer prevalence.

**Table 1:** The history of cancer.

**Time Period**	**Event/Discovery**	**Description**
**1600 BCE**	Edwin Smith Papyrus	The first identified example regards cancer, encompassing tumor explanations and surgical elimination strategies in ancient Egypt [29].
**460–370 BCE**	Hippocrates and “Karkinoma”	Hippocrates invented the term “karkinoma” (Greek for “crab”), assuming that cancer was induced by an asymmetry of bodily emotions [30].
**129–216 CE**	Galen's Differentiation	Galen developed Hippocrates' views, distinguishing between benign and malignant tumors, and developing early oncological concepts [31].
**Middle Ages**	Stagnation in Tumor Comprehending	Cancer treatment was dependent on superstitions and fundamental surgical techniques, with no substantial advances in understanding [32].
**17^th^ Century**	Innovation of the Microscope	The invention of the microscope enabled the exploration of cellular frameworks, advancing medical understanding of malignancies [33].
**1775**	Percivall Pott’s Soot Exposure Link	Pott discovered a link between chimney soot and scrotal malignancy in chimney sweeping, the first environmental cause related to cancer [34].
**19^th^ Century**	Surgeon Stephen Paget	Stephen made the initial deduction that cancer cells grow only in the organ in which they find compatibility, but eventually spread across all organs of the body through the circulation of blood. This paved the way for metastasis [35].
**1902**	Theodor Boveri’s Chromosomal Theory	Boveri argued that chromosomal aberrations were responsible for cancer development [36].
**1909**	Paul Ehrlich’s Immune Surveillance Theory	The Immune Surveillance Theory, proposed by Ehrlich, suggests that the immune system can identify and eliminate emerging tumor cells [37].
**1971**	National Cancer Act	President Nixon passed the National Cancer Act, setting in motion extensive financing for research and public education campaigns [38].
**1980s–Present**	Advances in Cancer Genetics and Treatment	Improvements in cancer treatment outcomes through discoveries in cancer genetics, targeted therapy, immunotherapy, and personalized medicine [39].

**Table 2:** World breast cancer report in 2023.

**Region**	**Incidence (millions)**	**Mortality (millions)**	**5-Year Prevalence (millions)**	**Survival Rate (%)**	**Mortality to Incidence Ratio**	**References**
Global	2.30	0.67	8.50	90%	0.29	[31]
Africa	0.23	0.13	0.75	70%	0.57	[32]
Americas	0.56	0.14	1.85	85%	0.25	[33]
Asia	1.07	0.30	3.65	75%	0.28	[31]
Europe	0.71	0.09	2.00	90%	0.13	[29]
Oceania	0.05	0.01	0.15	80%	0.20	[34]

**Table 3:** Bacterial involvement in promoting breast cancer, their sources, and mode of action.

**Source**	**Pathway**	**Key Bacteria**	**Role in Breast Cancer**	**Mechanism of Action**	**References**
Skin Microbiota	Migration from skin to breast tissue	*Staphylococcus epidermidis, Propionibacterium acnes*	Local inflammation, tissue irritation	Chronic inflammation-induced gene activation	[73]
Breastfeeding and Mammary Gland Microbiota	Introduction *via* infant oral flora and mammary ducts	*Lactobacillus, Staphylococcus aureus*	Protective or inflammatory (*e.g*., mastitis)	Immune modulation, inflammation	[74]
Gastrointestinal Tract and Gut Microbiome	Circulation of gut bacteria affecting breast tissue	*Estrobolome*	Estrogen metabolism, hormone-driven breast cancer risk	Estrogen modulation, immune system interaction	[10]
Oral Microbiome	Bacterial migration through the bloodstream	*Porphyromonas gingivalis, Fusobacterium nucleatum*	Chronic inflammation, immune response alteration	DNA damage, immune system suppression	[75]
Respiratory Tract Microbiome	Migration *via* the bloodstream from the lungs	Inhaled pathogens	Systemic inflammation, immune dysregulation	Chronic inflammation, gene expression alteration	[76]
Environmental and External Sources	Exposure to contaminated water, soil, and air	Environmental contaminants	Toxin production, oxidative stress	DNA damage, mutagenesis	[77]
Hormonal Imbalance and Systemic Inflammation	Estrogen metabolism imbalance, systemic inflammation	Disrupted microbiome influencing hormone levels	Promotes estrogen-driven breast cancer risk	Hormonal regulation, chronic immune activation	[78]

## LIST OF ABBREVIATIONS

BC: Breast Cancer
BFT: *B. Fragilis* Toxin
DNA: Deoxyribonucleic Acid
E. coli: Escherichia coli
H. pylori: Helicobacter pylori
WHO: World Health Organization
